# Heartland Virus Disease—An Underreported Emerging Infection

**DOI:** 10.3390/microorganisms12020286

**Published:** 2024-01-29

**Authors:** Zygmunt F. Dembek, Jerry L. Mothershead, Christopher M. Cirimotich, Aiguo Wu

**Affiliations:** 1 Battelle Memorial Institute, Support to DTRA Technical Reachback, Columbus, OH 43201, USA; dembek@battelle.org (Z.F.D.); cirimotichc@battelle.org (C.M.C.); 2 Applied Research Associates (ARA), Support to DTRA Technical Reachback, Albuquerque, NM 87110, USA; jmothershead@ara.com; 3 Defense Threat Reduction Agency (DTRA), Fort Belvoir, VA 22060, USA

**Keywords:** Heartland virus, Heartland, HRTV, bandavirus, RNA virus, tickborne disease, *Amblyomma americanum*, hemophagocytic lymphohistiocytosis, HLH, favipiravir, tanshinone

## Abstract

**Simple Summary:**

Heartland virus is an underrecognized emerging viral disease with potentially serious sequelae. The tick vector that transmits this disease, *A. americanum*, continues to expand to states and regions in the US where it was previously undocumented. This geographic expansion is stimulated by increasingly favorable climatic conditions and increasing numbers of host animal populations upon which the tick can feed. More work needs to be conducted to identify all aspects of the natural transmission cycle for HRTV. The true human population burden of Heartland virus is unknown, as comprehensive state, regional and national serosurveys have not been conducted. Expanded HRTV disease surveillance is needed. The absence of commercially available rapid and accurate HRTV tests dictates that the burden of testing remains with the CDC. Given the absence of antiviral treatment for Heartland virus disease, rest, fluids and OTC medications are most often used to treat patients, with hospital-provided IV fluids and supportive care for serious cases. Potential but unused treatments for HRTV infections include favipiravir, tanshinone I and IIa, anidulafungin and the NF-κB inhibitor SC75741, with anidulafungin potentially available for ‘off-label’ use for serious illness. Developmental research to create vaccines for SFTSV and HRTV suggests that vaccines might one day become available for prevention against HRTV infections.

**Abstract:**

First recognized 15 years ago, Heartland virus disease (Heartland) is a tickborne infection contracted from the transmission of Heartland virus (HRTV) through tick bites from the lone star tick (*Amblyomma americanum*) and potentially other tick species. Heartland symptoms include a fever <100.4 °F, lethargy, fatigue, headaches, myalgia, a loss of appetite, nausea, diarrhea, weight loss, arthralgia, leukopenia and thrombocytopenia. We reviewed the existing peer-reviewed literature for HRTV and Heartland to more completely characterize this rarely reported, recently discovered illness. The absence of ongoing serosurveys and targeted clinical and tickborne virus investigations specific to HRTV presence and Heartland likely contributes to infection underestimation. While HRTV transmission occurs in southern and midwestern states, the true range of this infection is likely larger than now understood. The disease’s proliferation benefits from an expanded tick range due to rising climate temperatures favoring habitat expansion. We recommend HRTV disease be considered in the differential diagnosis for patients with a reported exposure to ticks in areas where HRTV has been previously identified. HRTV testing should be considered early for those matching the Heartland disease profile and nonresponsive to initial broad-spectrum antimicrobial treatment. Despite aggressive supportive therapy, patients deteriorating to sepsis early in the course of the disease have a very grim prognosis.

## 1. Introduction

Heartland virus (HRTV) is a bandavirus first discovered in Missouri in 2009 and subsequently reported in 2012 [1]. HRTV causes Heartland virus disease (Heartland). This disease is known to be commonly transmitted by the lone star tick (*Amblyomma americanum*) in southern and midwestern US states [2], with most Heartland cases occurring in 14 US states [3]. Those infected with HRTV develop influenza-like symptoms and may become severely ill, potentially leading to death. There are currently no approved medications to treat the disease caused by HRTV infection, nor is there a vaccine available to prevent illness associated with HRTV infection. We examine the current literature on this virus, the illness it causes and related tick-borne diseases to better characterize the actual burden of human infection for HRTV, the potential for Heartland virus disease to geographically spread, its characteristics of infection and what viable potential antimicrobial therapies or vaccines are currently under development.

The disease is likely greatly underreported due to a lack of retrospective (serosurvey) and prospective (clinical and tickborne virus identification) investigations. The potential exists for a greatly expanded disease transmission range due to ecological changes in habitats favoring increases in tick vector populations and host access. A number of antimicrobials have shown promise in combatting HRTV infections in animals, but none are specifically approved for human use. Of these, favipiravir has undergone FDA clinical trials for influenza and COVID-19 treatment, but not for the treatment of patients with HRTV infection. Experimental vaccine development for another bandavirus, severe fever with thrombocytopenia syndrome virus (SFTSV), may demonstrate potential for HRTV vaccine development.

## 2. Virus Structure

HRTV belongs to the family *Phenuiviridae*, order *Bunyavirales*. HRTV is a tri-segmented negative-stranded RNA virus [4]. It contains three single-stranded RNA segments (L, M and S). The M segment of the virus encodes a polyprotein precursor that is cleaved into the glycoproteins Gn and Gc. Gc is a fusion protein enabling virus entry into host cells [5].

The genus bandavirus consists of nine tickborne bunyaviruses, with four known to cause disease in humans: Dabie bandavirus, also known as severe fever with thrombocytopenia syndrome virus (SFTSV), Banja bandavirus (BHAV), Guertu virus (GTV) and Heartland virus (HRTV) [4,6].

All bunyaviruses share a common genetic organization, with a segmented negative- or ambisense RNA genome composed of a small (S), medium (M) and large (L) genome segment. These segments encode structural proteins: the S segment encodes the nucleocapsid protein (N), the M segment encodes the virion glycoproteins (Gn) and (Gc) and the L segment encodes an RNA-dependent RNA polymerase (RdRp). Bunyaviruses can also encode non-structural proteins, in a negative- or positive-sense orientation, on the S segment (NSs) and/or the M segment (NSm) [7]. Bunyaviruses use a cap-snatching mechanism for viral mRNA transcription in which short-capped primers derived by the endonucleolytic cleavage of host mRNAs are used by the L-protein to transcribe viral mRNAs. The cap-snatching endonuclease of influenza virus is located in the N-terminal domain [8].

The overall structure of HRTV is similar to that of Dabie bandavirus, the severe fever with thrombocytopenia syndrome virus (SFTSV), which is also a member of the *Phenuiviridae* family [9]. SFTSV is a tick-borne phlebovirus that causes infections with similar symptoms to those caused by HRTV [10]. SFTSV is found in China, Japan and Korea, with respective approximate case–fatality rates (CFRs) of 6.2%, 27% and 23% [11]. There is a 27% and 38% difference in the respective viral RNA polymerase and N protein sequences between HRTV and SFTSV [12]. The CFR for HRTV infections has been observed to be between 5 and 10% of documented cases, with most of those succumbing also having serious underlying medical conditions [13].

Bhanja bandavirus (BHAV) is a tick-borne bunyavirus in the phlebovirus genus found in Africa and Eurasia [14]. It is associated with acute febrile illness and central nervous system involvement in humans. Bhanja virus infections have occurred from laboratory [15] and naturally acquired exposures [16], often via tickborne route of infection [17]. An additional pathogenic bandavirus is Guertu virus (GTV) [18], for which a serological survey among Chinese farmers and herdsmen found neutralizing antibodies, suggesting endemic human disease. GTV and SFTSV are serologically cross-reactive, and both are found in the same region of China. This indicates the need for laboratory tests with high specificity to optimally distinguish between tickborne viral infections [19].

## 3. HRTV in Mammals

A 2013 convenience sample of wild mammals in various states found HRTV antibodies in the states of Florida, Georgia, Illinois, Indiana, Kansas, Kentucky, Missouri, North Carolina, Tennessee, Texas, New Hampshire, Maine and Vermont. Seropositivity for HRTV has been noted in the following wild mammal species: white-tailed deer (*Odocoileus virginianus*), coyote (*Canis latrans*), raccoon (*Procyon lotor*) and moose (*Alces alces*) [20], and also in Virginia opossums (*Didelphis virginiana*) and horses (*Equus caballus*) [21].

Table 1 below indicates the four mammalian species that tested seropositive for HRTV and in which US state they were located in a wildlife serosurvey [20]:

Attempts to experimentally infect mice, rabbits, hamsters, chickens, raccoons, goats and deer with HRTV have failed to produce detectable viremia [9]. However, Ag129 mice {interferon-α/β/γ receptor-deficient [Ag129]} have been found to be an ideal in vitro model for dose-dependent HRTV viremia and associated illness and death [21]. The interferon deficiency characteristic of the Ag129 mice enables their susceptibility to a wide range of viruses [22], and they are also widely used in dengue vaccine research [23].

## 4. Human Case Distribution and Range

A 2013–2017 survey of 85 individuals ≥12 years of age for whom clinicians contacted state health agencies for HRTV testing found 16 (19%) had acute HRTV infection, 1 (1%) had a past infection and 68 (80%) had no infection. Those with HRTV infection resided in seven states; 12 (75%) were male, and the median age (range) was 71 (43–80) years. Most cases reported fatigue, anorexia, nausea, headache, confusion and arthralgia or myalgia. The illness onset occurred from April through September, with 14 cases (88%) hospitalized and 2 (13%) deaths. Fourteen (88%) participants reported a tick discovery on themselves within 2 weeks prior to the illness onset. The HRTV-infected individuals were significantly older (*p <* 0.001) and more likely to report an attached tick (*p =* 0.03) than the uninfected individuals [24]. Heartland cases often demonstrate preexisting comorbidities, including time spent outdoors (e.g., camping, farming or hunting), along with a history of tick exposure [13].

Tickborne bacterial and protozoan human disease cases doubled in the US between 2004 and 2016 [25]. Although >60 Heartland virus human cases have been reported to date, it is assumed that the actual number of cases is significantly larger. Human cases have been reported from Arkansas, Georgia, Illinois, Indiana, Iowa, Kansas, Kentucky, Missouri, New York, North Carolina, Oklahoma, Tennessee and Virginia, and deaths from HRTV infections have occurred in Missouri, Tennessee, Oklahoma and Virginia [3]. Given the Table 1 demonstrating that HRTV seropositive mammals were found in the states of Georgia, Illinois, Indiana, Kansas, Kentucky, North Carolina, Tennessee and Virginia, it is anticipated that human infections would also occur in these states, and seropositive wild mammals would be found upon inspection in the states of Arkansas, Iowa, New York and Oklahoma.

It is notable that the HRTV virus seroprevalence in blood donors in a 2013 convenience sample taken in Missouri was 0.9% [26]. Given the 2013 population of Missouri (6.043 million), this then equates to a potential estimated statewide total of 543,870 Heartland virus cases! It therefore appears likely that the reported Heartland virus cases are a small fraction of those that occur annually in Missouri, and perhaps elsewhere as well. It follows that the overall Heartland virus disease case numbers are much larger nationally than are now recognized.

A recent study collecting and analyzing ticks near where two Heartland virus cases had occurred in Illinois revealed HRTV-positive pools of adult male ticks at two locations over 270 miles apart. This suggests widespread HRTV-infected tick populations in Illinois [27]. It is also likely that HRTV is now present in many other states where human infections are not suspected or tested. Besides the states mentioned above, wild mammals have been found to be infected with HRTV, but human cases have not been identified in the states of Florida, Texas, New Hampshire, Maine and Vermont. Additionally, states where the presence of *A. americanum* has been documented but that are not included in the above compilation may also be considered at risk for Heartland Virus transmission. This list includes most states east of longitude 110° W, i.e., every US state east of Texas, Oklahoma, Kansas, Nebraska, Iowa and Illinois (to include these states), for a total of 31 of the 48 continental states where this tick has been identified to date [28].

## 5. Tick Attachment

*A. americanum* is considered a hunter tick and will crawl across many meters when attracted by a host’s scent [29]. The behavior that ticks engage in to feed on passing animals is known as questing. While engaging in this activity, ticks hold onto leaves and grass by their third and fourth pairs of legs and hold their first pair of legs outstretched, awaiting contact with a potential host. When a potential host brushes against the location where a tick is waiting, it rapidly climbs aboard the bird or mammal [30]. Further, the larvae of *A. americanum* exhibit questing behavior in the central US in the late summer. Elevated temperatures and low relative humidity are known to extend the larval hardening period for this species from 10 to 29 days [31].

Transstadial transmission (the transmission of HRTV throughout the development of tick life stages) from larvae to nymphs and then to adults has been documented [32]. Vertical transmission of HRTV also occurred in the progeny of infected females [32]. A recent study of human attachment site preferences for ticks native to New York states that *A. americanum* has a preference for attachment to the thighs, groin and pelvic areas. *A. americanum* can also transmit *Ehrlichia chaffeeensis* and *E. ewingii* and is associated with Southern Tick-borne Rash-Associated Illness (STARI) [33]. *A. americanum* also transmits the Bourbon and Tacaribe viruses, along with *Rickettsia parkeri* and *Franciscella tularensis* [34].

## 6. Symptoms

The incubation period from a tick bite to symptom onset ranges from a few days to 2 weeks. The signs and symptoms of Heartland virus disease (Heartland) are often similar to those of other tickborne diseases, e.g., ehrlichiosis and anaplasmosis. These symptoms include a fever <100.4 °F, lethargy, fatigue, headaches, myalgia, loss of appetite, nausea, diarrhea, weight loss, arthralgia, leukopenia and thrombocytopenia. Elevated liver transaminases may also be present [24,35].

Immunocompromised individuals are at particular risk from HRTV infection, as has occurred with heart transplant recipients [36] and others who developed hemophagocytic lymphohistiocytosis (HLH) as a consequence of HRTV infection [37]. HLH can be a life-threatening sequela from HRTV infection [38,39,40]. With HLH, histiocytes and lymphocytes (white blood cells) attack other blood cells, and abnormal blood cells accumulate in the liver and spleen. HLH can cause death in weeks or months even if treated [39]. Supportive care in the form of packed red blood cells and platelet transfusions for platelets with nadir of 73 × 10^9^/L has been used successfully in a single case [36].

## 7. HRTV Laboratory Testing

The HRTV real-time reverse transcription polymerase chain reaction (RT-PCR) laboratory test provides qualitative detection of HRTV RNA from clinical samples (serum and CSF) obtained from individuals with a suspected infection. HRTV RNA in clinical specimens is extracted, and RT-PCR testing is performed, employing oligonucleotide forward and reverse primers and a TaqMan^®^ hydrolysis probe specific to the small segment of the non-structural protein region of HRTV. The presence of HRTV RNA in a specimen can be used to support the diagnosis of acute HRTV infection [41,42].

## 8. Recent Analytical Improvements

IgM and IgG microsphere immunoassays (MIAs) have recently been developed to test sera for HRTV and to distinguish between recent and past infections. This method enables HRTV antigens to become attached to anti-HRTV monoclonal antibodies covalently bound to microspheres. Human sera antibodies react with microsphere complexes and are detected using a BioPlex^®^ 200 instrument (BioRad, Hercules, CA, USA). The sensitivities, specificities and accuracies of the IgM and IgG MIAs were all >95%. HRTV IgM and IgG MIAs are accurate and rapid methods to serologically identify recent and past HRTV infections [43].

## 9. Diagnosis

The diagnosis of HRTV infection is made by examining a patient for signs and symptoms, obtaining a personal history of living in tick-frequented areas where Heartland virus is found and asking about possible tick exposure, along with positive blood tests. Other infectious diseases, including anaplasmosis and ehrlichiosis, can be ruled out if doxycycline treatment has had no effect. Further, antibody titers and RT-PCR may be used to detect HRTV RNA in the blood. There are no commercially available tests for Heartland virus, and so most molecular and serologic HRTV testing is conducted by the CDC as per above [44].

## 10. Treatment

There are no approved antimicrobial medications for the prevention or treatment of Heartland virus infection. Rest, fluids and over-the-counter (OTC) pain medications may alleviate some symptoms of infection. Hospitalization may be required for IV fluids and supportive care [35]. High-dose corticosteroid treatment is the primary means of HLH treatment, often complemented with immunoglobulins, the topoisomerase II inhibitor Etoposide, the interleukin-I receptor antagonist Anakinra or the kinase inhibitor Ruxolitinib [45].

## 11. Additional Inferences—Symptoms, Testing, Diagnosis and Treatment

From patients with confirmed positive tests for HRTV, we know that the Heartland disease syndrome presents symptoms similar to those of other tickborne diseases. The differential diagnosis for Heartland includes other regionally endemic tick-borne diseases, such as human monocytotropic ehrlichiosis (*Ehrlichia chaffeensis*) and related ehrlichial agents, Rocky Mountain Spotted Fever (*Rickettsia rickettsii*), human granulocytic anaplasmosis (*Anaplasma phagocytophilum*), Lyme disease (*Borrelia burgdorferi*), Bourbon virus and potentially Powassan, Colorado tick fever and SFTS viruses, with differential diagnosis determination aided by clinical presentation and travel history [13]. HRTV-infected immunocompromised patients and those developing HLH are at risk of death, even with treatment. HRTV confirmatory testing is likely not performed unless specifically requested by a clinician. As most such testing is performed at the CDC rather than through routine hospital laboratory testing, this may add an additional complication for disease determination for many clinicians. The absence of readily available viable treatment medications also adds to the burden of adverse outcomes from Heartland disease.

## 12. Heartland Case Studies

With a paucity of reports on HRTV reported in peer-reviewed journals, healthcare providers may not have a high index of suspicion for such cases in everyday practice. A review of three case reports (below) on fatal illness due to HRTV infection may be illustrative. All three cases involved males over the age of 60, with comorbidities ranging from mild to severe, who resided in states in which HRTV had been identified. All three had the potential, through work or residence, to be exposed to ticks, and, indeed, in two cases, exposure to a recent (~2 week) tick bite was reported. In two cases, the initial presentation involved fever, malaise and general non-specific symptoms. The third patient presented with mental status changes. The initial diagnostics were normal or slightly abnormal, with leukopenia, thrombocytopenia and elevated transaminases being the most common findings. The diagnosis of a tick-borne disease was entertained, and all three were initially placed on doxycycline. Two patients, with milder symptoms, were discharged after initial treatment but returned with worsening symptoms, including mental status changes, within a few days.

All the patients rapidly deteriorated to septic shock within a few days of the initial evaluation. Once admitted, all the patients received extensive diagnostic evaluations that involved full batteries of hematological studies, radiological evaluations, including CT or MRI scans and blood and fluid cultures. Serologies for common viruses and tick-borne pathogens were ordered, with negative results.

Although doxycycline was used as an initial therapy, as the patients’ conditions worsened, other broad-spectrum antibiotics were empirically added. Other treatments were supportive, including, as necessary, assisted ventilation, platelet transfusions and other reactive treatments for worsening sepsis. Despite these measures, they all progressed to end-organ failure (liver, kidneys) and disseminated intravascular coagulopathy as precursors to their demise, which occurred within two weeks of presenting illnesses.

All definitive diagnoses of HRTV infection were made postmortem as the result of reverse transcriptase PCR testing for HRTV mRNA.

The lessons learned from these cases can be summed up as the following:

1. HRTV disease should be considered in the differential diagnosis of anyone who has a reported exposure to ticks in an area in which HRTV has been previously identified;
2. Testing for HRTV should be considered early for those matching the profile who are not positively responsive to initial broad-spectrum treatment;
3. Despite aggressive supportive therapy, those who deteriorate to sepsis early in the course of the disease have a very grim prognosis.

### 12.1. Case 1: Male, 80 Years Old—History of Chronic Obstructive Pulmonary Disease and Heavy Alcohol Use—Tennessee, July 2013

#### 12.1.1. Physical Findings

He was hospitalized with fever, confusion, leukopenia and thrombocytopenia and developed multiorgan failure and hemorrhage. He has a history of multiple falls during the prior week, an altered mental state, fevers, decreased appetite, a dark stool and multiple tick bites. He had a fever of 100 F upon presentation at the hospital. On hospital day 2, he was transferred to a quaternary care center for persistent delirium and worsening tachypnea. He was admitted to the hospital ICU within hours for worsening hypoxia and hypotension with concern for sepsis, with a fever of 103.6 F and purpura. His clinical findings worsened during hospitalization, including worsening thrombocytopenia, leukopenia, decreasing Hgb and increased creatine kinase, AST, ALD, LDH and creatinine. He eventually developed respiratory failure, a troponin leak, acute kidney failure and upper gastrointestinal bleeding [40].

#### 12.1.2. Laboratory Results

He tested negative for ehrlichiosis. Upon presentation at the hospital, the patient had hyponatremia, elevated aspartate aminotransferase and alanine aminotransferase, leukopenia and thrombocytopenia. The Hgb level and prothrombin time were normal. A CT of his chest and head showed no abnormalities. He was admitted with suspected ehrlichiosis [40].

Immunohistochemical assays for *Ehrlichia*, *Anaplasma*, the spotted fever group *Rickettsia* and *Leptospira* species were all negative. RNA was extracted from premortem blood and serum samples, as well as fixed postmortem spleen and lymph tissues. Qualitative RT-PCR for HRTV RNA was conducted. HRTV antigens were detected in the postmortem spleen and lymph nodes by immunohistochemistry, and HRTV was detected in premortem blood by reverse transcription polymerase chain reaction and by isolation in a cell culture [40].

#### 12.1.3. Treatment and Outcome

He was given doxycycline upon hospital admission and died on hospital day 15 [40].

### 12.2. Case 2: Male, 68 Years—Past Medical History of Limited Intracerebral Hemorrhage, Stage T2b Melanoma and Hypertension—Tennessee, July 2015

#### 12.2.1. Physical Findings

He presented to the hospital ER with complaints of a rash and a pain in his left lower extremity. He reported a tick bite during the 2 weeks preceding the onset of the illness. He later developed a fever, progressive weakness, recurrent falls, nausea, vomiting and confusion and returned to the ER 4 days later, where he was admitted to the hospital. Day 5 hospital: he was transferred to a medical center for septic shock, altered mental status and acute renal failure; he became tachycardic and hypotensive despite treatment. He developed severe septic shock, acute respiratory distress syndrome, disseminated intravascular coagulation, renal failure, atrial fibrillation with rapid ventricular response and delirium [46].

#### 12.2.2. Laboratory Results

Normal findings were documented at the initial ER visit. Upon return to the ER, thrombocytopenia, hyponatremia, elevated aspartate aminotransferase and alanine aminotransferase were documented. Day 2 hospital: he had normal CSF and worsening hyperbilirubinemia. Day 5 hospital: he had leucocytosis, thrombocytopenia, coagulopathy, increased creatine kinase and lactic dehydrogenase, elevated creatinine, severe anon-gap metabolic acidosis and increased ferritin. Blood, urine and CSF cultures: No bacterial growth was found. Serologies for *Borrelia burdorferi*, *Ehrlichia* sp. and *Rickettsia* sp. were all negative. He was negative for HIV and hepatitis A, B and C [46].

#### 12.2.3. Treatment and Outcome

He was treated with doxycycline. Upon his return to the hospital, he was treated with iv vancomycin and ceftriaxone. He died on hospital day 6 [46].

### 12.3. Case 3: Male, Late 60s—History of Splenectomy from Remote Trauma, Coronary Artery Disease and Hypertension—Maryland and Virginia, November 2021

#### 12.3.1. Physical Findings

He was seen in the hospital ED after 5 days of fever, diarrhea, dyspnea, myalgias and malaise. He was admitted on day 7 with confusion, an unsteady gait, fecal and urinary incontinence, progressive encephalopathy, hyponatremia and rising transaminases; the neurologic workup and imaging were unremarkable, and the CT scan showed new pelvic and inguinal lymphadenopathy. With continued clinical deterioration, the patient was transferred to a tertiary care center with fatigue and disorientation. He then demonstrated new hepatomegaly and lower extremity livedo reticularis. He then had respiratory failure, renal failure and cardiac arrest [38].

#### 12.3.2. Laboratory Results

Initially, he was found to have hyponatremia, mildly elevated liver enzymes, leucopenia and thrombocytopenia. At the tertiary care center, he was then found to have elevated creatine kinase, lactate, lactate dehydrogenase, ferritin and the interleukin 2 receptor [38].

#### 12.3.3. Treatment and Outcome

He was initially administered doxycycline. Upon hospital admission, he was treated with hypertonic saline, iv doxycycline and piperacillin/tazobactam. He died on day 13 after symptom onset [38].

## 13. Additional Inferences—Heartland Case Studies

This review of three fatal Heartland case reports indicates that potential risk factors leading to death from HRTV infection include male gender, age over 60 years, the presence of additional mild-to-severe medical conditions and a history of exposure to tick bites. The initial case presentation with non-specific symptoms did not provide sufficient information for disease recognition. In geographic areas where HRTV has previously been identified, the HRTV testing of suspect patients with exposure to ticks should be prioritized. All three cases were initially treated with doxycycline, which is the treatment of choice (along with tetracycline) for the tickborne diseases ehrlichiosis, Lyme disease, Rocky Mountain spotted fever and relapsing fever. While it is recommended that such medical treatment not be delayed for patients with clinical findings suggestive of tick-borne disease [47], this therapy has been found ineffective for Heartland disease. With the absence of a positive response to initial broad-spectrum antimicrobial treatment and/or a worsening medical condition, HRTV testing should be initiated if it has not already been conducted. Given the absence of a specific treatment for Heartland disease, the discovery of a viable therapy for this disease should be a priority.

## 14. Tick Infection, Distribution and Range

A 2016 study discovered more than 8000 single-nucleotide polymorphisms in 90 *A. americanum* ticks from five locations in Maine, North Carolina, New York, Oklahoma and South Carolina. The newly established tick populations in New York and Oklahoma were as genetically diverse as the historic range populations in both North and South Carolina. However, substantial population structure occurs among regions, such that new populations in New York and Oklahoma are genetically distinct from historic range populations and from one another. Ticks from a laboratory colony are genetically distinct from wild populations, which is a key factor to consider when conducting disease transmission studies [48].

*A. americanum* may become infected with HRTV at the larval, nymphal and adult stages of its life cycle [9,32]:

- Adult ticks may feed on an infected animal and may pass the virus vertically to tick larvae.
- Larvae feed on mammalian hosts and transmit HRTV or become infected while co-feeding with infected ticks.
- Larvae pass HRTV to nymphal-stage ticks. The transfer of parasites (or in this case, a virus) is referred to as transstadial passage.
- Nymph and adult ticks infected transstadially or by co-feeding transmit HRTV to humans or mammalian hosts.
- Co-feeding or transstadial transmission could occur from nymphal- to adult-stage ticks.
- Overwintering or infected nymphal or adult stages could occur, with possible transmission in the spring.

A recent study subjected *A. americanum*, *Dermacentor variabilis* and *Ixodes scapularis* to varying temperatures and humidity levels simulating climatic stress and found that, in terms of survival rates regarding water loss, *I. scapularis* was most susceptible when compared to *A. americanum* and *D. variabilis* [49].

The apparent absence of the lone star tick in the New England states may indicate that other tick species may carry the HRTV virus. One potential candidate for this with subsequent HRTV transmission is the Asian longhorned tick (*Haemaphysalis longicornis),* which has been shown to acquire and transmit HRTV in the laboratory [50]. The techniques used to experimentally transmit HRTV in *H. longicornis* ticks could also be attempted using other ticks to help determine their potential for HRTV transmission, including *I. scapularis* and *D. variabilis*. In consideration of the fact that changing climactic conditions may foster further spread of tickborne HRTV, other global tick species capable of disease transmission could similarly be tested [51,52,53].

*A. americanum* is found primarily in the south, southeast and eastern coast of the US, with scattered observations in more northerly states of the upper midwest and northeast and the lower areas of Ontario [54]. It is important to realize that these observations primarily reflect recent tick surveillance data. Where a state or region lacks robust tick surveillance, tick species identification cannot occur.

The range of the lone star tick *A. americanum* appears to continue to expand northward in the US. A New Jersey study on passive tick surveillance conducted from 2006 to 2016 demonstrated that *A. americanum* increased significantly over that time. By 2016, *A*. *americanum* had expanded northward in Monmouth County and accounted for nearly half (48.1%) of submissions [55]. *A. americanum* is present in the southern portions of New York and dominant on Long Island, where it also feeds aggressively on deer, medium-sized animals and birds.

Climate data collected from meteorological stations in the United States and Canada have been used to estimate the minimum survival temperatures for *A. americanum*. Based on this predictive model, this tick’s range is predicted to continue to increase into the northern US states and southern Quebec and Ontario in Canada [56]. Future climate modeling indicates that other areas in the Americas, Europe and Asia may prove suitable for supporting *A. americanum.* Specifically, the following geographic areas may support lone star tick geographic expansion: the Bahamas, the southeast of Canada and the northeast of Mexico in North America; Switzerland, the southern part of France, Italy, Austria, Germany, Andorra, Croatia and Serbia in Europe; and the southeastern part of China, South Korea and Japan in Asia [57].

Rising global temperatures, ecological changes, reforestation and increases in commerce and travel are all important contributors to the range expansion of ticks and tickborne pathogens. Lone star ticks primarily feed on mammals and birds. Their population growth is inherently linked to increasing populations of coyotes, deer and wild turkeys. The climatic conditions now favor the establishment and expansion of lone star ticks along the southern New England coast. Abundant reproductive hosts, accommodating climatic conditions and the genetic adaptability of the lone star tick support the continued invasion and establishment of this tick in the Northeast. *A. americanum* may eventually displace local tick species and alter tickborne disease patterns by transmitting different pathogens than those prior species [58].

## 15. Potential Treatments for HRTV Infection

A recent study used a mouse model to test antiviral agents against HRTV infections. The survival rates for the HRTV-infected mice with favipiravir and ribavirin orally administered 5 days following a lethal inoculation of HRTV were 100% and 33%, respectively. All mice that were similarly administered solely with a methylcellulose solvent died following infection. This study indicates the potential for favipiravir as an antiviral candidate for the treatment of patients with HRTV infection [59]. Unfortunately, although favipiravir is used to treat influenza in Japan and has undergone Phase II and Phase III FDA clinical trials for influenza [60] and COVID-19 treatment [61], it remains unapproved for human use by the US FDA [62].

Recent research has identified tanshinone I as a cap-dependent endonuclease (CEN) inhibitor with broad-spectrum antiviral effects on negative-stranded, segmented RNA viruses, including bandavirus, orthomyxovirus and arenavirus. CEN mediates the critical cap-snatching step of viral RNA transcription [63]. For example, cap-snatching with influenza virus occurs when, during the transcription of viral mRNA, the ribonucleoprotein complex steals short, 5′-capped transcripts produced by cellular DNA-dependent RNA polymerase II (RNAPII) and uses them to prime the transcription of viral mRNA [64].

Both tanshinone I and IIa were found to effectively inhibit HRTV in vitro with EC50s at the micromolar level. It has been hypothesized that tanshinone broad-spectrum antiviral activity may be due to the targeting of endonuclease to employ antiviral effects [65]. Tanshinone IIa is a potential therapeutic for ischemic stroke that has been successful in pre-clinical rodent studies but has shown inconsistent efficacy results in human patients [66].

The antifungal drug anidulafungin is an amphiphilic hexapeptide linked to an alcoxytriphenyl side chain. Anidulafungin belongs to a class of antifungals known as echinocandins, as they are derived from echinocandin B_0_, which is produced by *A. nidulans* [67]. Anidulafungin’s mechanism of action is based on the inhibition of β-(1,3)-D-glucan synthesis that forms the fungal cell wall [68]. Recent research has demonstrated that HRTV (along with other viruses) was inhibited by anidulafungin in a dose-dependent manner by interfering with the virus’s entry into cells. Anidulafungin is approved by the FDA for antifungal use [69].

The Nuclear Factor Kappa B (NF-κB) inhibitor SC75741 is a novel antiviral against emerging tick-borne bandaviruses and has been shown to reduce HRTV viral synthesis in vitro [70]. The green tea polyphenols (-)-epigallocatechin gallate (EGCg) and (-)-epigallocatechin (EGC) have been found to be effective antivirals against SFTSV and could also prove effective against HRTV [71].

In sum, there are a handful of experimental antiviral compounds that can be further advocated for research against HRTV infections. Of these, anidulafungin is FDA-approved for antifungal use but not for antiviral properties. It could potentially be used in severe HRTV infection cases as an “off-label” drug [72].

## 16. Vaccine Development

Vaccine development is being explored for SFTS, caused by SFTSV. Vaccine candidates include live-attenuated virus-based, viral vector-based and DNA-based vaccines. To date, vaccines have been tested in mice, hamsters, ferrets and cats. Non-human primate models of SFTS have been attempted but are not yet established, indicating that the background work required for human vaccine trials has yet to occur. Because SFTSV is closely related to HRTV (both are bandaviruses), a similar vaccine development for Heartland virus may be possible [73]. An exploratory study using macaques found that exposures to a 10^6^ TCID_50_ dose of both viruses did not cause serious illness in these animals [74]. This research indicates that the development of a similar vaccine candidate for HRTV may be possible.

A computational analysis has recently been used to create multi-epitope subunit vaccines (MEVCs) specific to HRTV. These hypothetical vaccines contain protein-specific and proteome-wide helper T-cell lymphocytes (HTL), linear B cell and cytotoxic T-cell (CTL) epitope mapping combined with suitable linkers. Four vaccines were created from nucleocapsid protein, replicase, glycoprotein and whole-proteome-wide constructs, and they demonstrated stronger antigenic and non-allergenic behavior, good binding with toll-like receptor 7 (TLR7) and antigen neutralization via antibody production [75]. This computational assessment provides a pragmatic background from which future live vaccine experiments may be conducted. Towards this goal, recent experiments have used a recombinant vaccinia virus (VACV) expression system to produce arbovirus virus-like particles (VLPs) that can be used as subunit or vectored vaccines [76].

## Figures and Tables

**Table 1:** Convenience sample of wild mammals seropositive for HRTV antibodies from 2013. State and species discovered.

State	Species
Florida	White-tailed deer (*Odocoileus virginianus*)
Georgia	White-tailed deer
Illinois	Coyote (*Canis latrans*)
gIndiana	Raccoon (*Procyon lotor*)
Kansas	Coyote
Kentucky	Raccoon
Missouri	Raccoon
New Hampshire	Moose (*Alces alces*), White-tailed deer
North Carolina	White-tailed deer
Tennessee	Raccoon
Texas	Raccoon
Vermont	White-tailed deer
Virginia	Raccoon
West Virginia	Raccoon

## Data Availability

Data are contained within the article.

## References

[1] Esguerra E.M. (2016). Heartland Virus: A New Virus Discovered in Missouri. Mo. Med..

[2] Reynolds E.S., Wooldridge J.T., Stevenson H.L., Thangamani S. (2023). The Lone Star tick, *Amblyomma americanum*, salivary factors exacerbate the clinical outcome of Heartland virus disease in a small animal model. Sci. Rep..

[3] Centers for Disease Control and Prevention (2022). Statistics and Maps. Centers for Disease Control and Prevention. https://www.cdc.gov/heartland-virus/statistics/index.html.

[4] International Committee on Taxonomy of Viruses (2024). Master Species Lists. ICTV_Master_Species_List_2022_MSL38.v3.xlsx. https://ictv.global/msl.

[5] Shah T., Li Q., Wang B., Xia X. (2023). Geographical distribution and pathogenesis of ticks and tick-borne viral diseases. Front. Microbiol..

[6] Kuhn J.H., Abe J., Adkins S., Alkhovsky S.V., Avsic-Zupanc T., Ayllon M., Bahl J., Balkema-Buschmann A., Ballinger M.J., Baranwall V.K. (2023). 2023. Annual (2023) taxonomic update of RNA-directed RNA polymerase-encoding negative-sense RNA viruses (realm *Rhiboviria*: Kingdom *Orthonavirae*: Phylum *Negarnaviricota*). J. Gen. Virol..

[7] Fares M., Brennan B. (2022). Virus-host interactions during tick-borne bunyavirus infection. Curr. Opin. Virol..

[8] Reguera J., Weber F., Cusack S. (2010). Bunyaviridae RNA Polymerases (L-Protein) Have an N-Terminal, Influenza-Like Endonuclease Domain, Essential for Viral Cap-Dependent Transcription. PLoS Pathog..

[9] Brault A.C., Savage H.M., Duggal N.K., Eisen R.J., Staples J.E. (2018). Heartland Virus Epidemiology, Vector Association, and Disease Potential. Viruses.

[10] Wormser G.P., Pritt B. (2015). Update and Commentary on Four Emerging Tick-Borne Infections Ehrlichia muris–like Agent, Borrelia miyamotoi, Deer Tick Virus, Heartland Virus, and Whether Ticks Play a Role in Transmission of *Bartonella henselae*. Infect. Dis. Clin. N. Am..

[11] Casel M.A., Park S.J., Cusack S. (2021). Severe fever with thrombocytopenia syndrome virus: Emerging novel phlebovirus and their control strategy. Exp. Mol. Med..

[12] Brennan B., Li P., Zhang S., Li A., Liang M., Li D., Elliott R.M. (2015). Reverse genetics system for severe fever with thrombocytopenia syndrome virus. J. Virol..

[13] Mantlo E.K., Haley N.J. (2023). Heartland virus: An evolving story of an emerging zoonotic and vector-borne disease. Zoonotic Dis..

[14] Swei A., Russell B.J., Naccache S.N., Kabre N., Veeraraghavan N., Pilgard M.A., Johnson B.J.B., Chiu C.Y. (2013). The genome sequence of Lone Star Virus, a highly divergent Bunyavirus found in the *Amblyomma americanum* tick. PLoS ONE.

[15] Calisher C.H., Goodpasture H.C. (1975). Human infection with Bhanja virus. Am. J. Trop. Med. Hyg..

[16] Vilibik-Cavlek T., Stevanovic V., Krcmar S., Savic V., Kovac S., Bogdanic M., Maljkovic M.M., Sabadi D., Santini M., Potocnik-Hunjadi T. (2023). Detection of Bhanja Bandavirus in Patients with Neuroinvasive Disease of Uknown Etiology in Croatia. Microorganisms.

[17] Hubalek Z. (2009). Biogeography of Tick-Borne Bhanja Virus (*Bunyaviridae*) in Europe. Interdiscip. Perspect. Infect. Dis..

[18] Shen S., Duan X., Wang B., Zhu L., Zhang Y., Zhang J., Wang J., Luo T., Kou C., Liu D. (2018). A novel tickborne phlebovirus, closely related to severe fever with thrombocytopenia syndrome virus and Heartland virus, is a potential pathogen. Emerg. Microbes Infect..

[19] Mendoza C.A., Ebihara H., Yamaoka S. (2019). Immune modulation and immune-mediated pathogenesis of emerging tickborne Bayangviruses. Vaccines.

[20] Riemersma K.K., Komar N. (2015). Heartland Virus Neutralizing Antibodies in Vertebrate Wildlife, United States, 2009–2014. Emerg. Infect. Dis..

[21] Bosco-Lauth A.M., Calvert A.E., Root J.J., Gidlewski T., Bird B.H., Bowe R.A., Muehlenbachs A., Zaki S.R., Brault A.C. (2016). Vertebrate host susceptibility to Heartland virus. Emerg. Infect. Dis..

[22] van den Broek M.F., Müller U., Huang S., Zinkernagel R.M., Aguet M. (1995). Immune defence in mice lacking type I and/or type II interferon receptors. Immunol. Rev..

[23] Sarathy V.V., Milligan G.N., Bourne N., Barrett A.D. (2015). Mouse models of dengue virus infection for vaccine testing. Vaccine.

[24] Staples J.E., Pastula D.M., Panella A.J., Rabe I.B., Kosoy O.I., Walker W.L., Velez J.O., Lambert A.J., Fischer M. (2020). Investigation of Heartland Virus Disease Throughout the United States, 2013–2017. Open Forum Infect. Dis..

[25] Rosenberg R., Lindsey N.P., Fischer M., Gregory C.J., Hinckley A.F., Mead P.S., Paz-Bailey G., Waterman S.H., Drexler N.A., Kersh G.J. (2018). Vital Signs: Trends in Reported Vectorborne Disease Cases—United States and Territories, 2004–2016. MMWR Morb. Mortal. Wkly. Rep..

[26] Lindsey N.P., Menitove J.E., Biggerstaff B.J., Turabelidze G., Parton P., Peck K., Basile A.J., Kosoy O.I., Fischer M., Staples J.E. (2019). Seroprevalence of Heartland virus antibodies in blood donors, northwestern Missouri, USA. Emerg. Infect. Dis..

[27] Tuten H.C., Burkhalter K.L., Noel K.R., Hernandez E.J., Yates S., Wojnowski K., Hartleb J., Debosik S., Holems A., Stone C.M. (2020). Heartland virus in humans and ticks, Illinois, USA, 2018–2019. Emerg. Infect. Dis..

[28] Centers for Disease Control and Prevention (2021). Lone Star Tick Map. Centers for Disease Control and Prevention. https://www.cdc.gov/ticks/maps/lone_star_tick.pdf.

[29] Sonenshine D.E. (2018). Range Expansion of Tick Disease Vectors in North America: Implications for Spread of Tick-Borne Disease. Int. J. Environ. Res. Public Health.

[30] Centers for Disease Control and Prevention (2020). How Ticks Spread Disease. https://www.cdc.gov/ticks/life_cycle_and_hosts.html.

[31] Leal B., Zamora E., Fuentes A., Thomas D.B., Dearth R.K. (2020). Questing by Tick Larvae (Acari: Ixodidae): A Review of the Influences That Affect Off-Host Survival. Ann. Entomol. Soc. Am..

[32] Godsey M.S., Savage H.M., Burkhalter K.L., Bosco-Lauth A.M., Delorey M.J. (2016). Transmission of Heartland Virus (Bunyaviridae: *Phlebovirus*) by Experimentally Infected *Amblyomma americanum* (Acari: Ixodidae). J. Med. Entomol..

[33] Hart C., Schad L.A., Bhaskar J.R., Reynolds E.S., Morley C.P., Thangamani S. (2022). Human attachment site preferences of ticks parasitizing in New York. Sci. Rep..

[34] Kennedy A.C., Marshall E. (2021). Lone Star Ticks (*Amblyomma americanum*): An Emerging Threat in Delaware. Del. J. Public Health.

[35] Centers for Disease Control and Prevention (2022). Symptoms, Diagnosis and Treatment. Heartland Virus Disease (Heartland). https://www.cdc.gov/heartland-virus/symptoms-treatment/index.html.

[36] Hevey M.A., O’Halloran J.A., Jagger B.W., Staples J.E., Lambert A.J., Panella A.J., Kosoy O.I., Turabelidze G., Raymer D.S., Ewald G.A. (2019). Heartland virus infection in a heart transplant recipient from the Heartland. Transpl. Infect. Dis..

[37] Carlson A.L., Pastula D.M., Lambert A.J., Staples J.E., Muehlenbachs A., Turabelidze G., Eby C.S., Keller J., Hess B., Buller R.S. (2018). Heartland Virus and Hemophagocytic Lymphohistiocytosis in Immunocompromised Patient, Missouri, USA. Emerg. Infect. Dis..

[38] Liu S., Kannan S., Meeks M., Sanchez S., Girone K.W., Broyhill K.C., Martines R.B., Bernick J., Flammia L., Murphy J. (2023). Fatal Case of Heartland Virus Disease Acquired in the Mid-Atlantic Region, United States. Emerg. Infect. Dis..

[39] Gotwals J., Sather R., Gersten T. (2023). Acquired Hemaphagocytic Lymphohistiocytosis. Health Encyclopedia. University of Rochester Medical Center. https://www.urmc.rochester.edu/encyclopedia/content.aspx?ContentTypeID=134&ContentID=212.

[40] Muehlenbachs A., Fata C.R., Lambert A.J., Paddock C.D., Velez J.O., Blau D.M., Staples J.E., Karlekar M.B., Bhatnagar J., Nasci R.S. (2014). Heartland virus-associated death in Tennessee. Clin. Infect. Dis..

[41] Savage H.M., Godsey M.S., Lambert A., Panella N.A., Burkhalter K.L., Harmon J.R., Lash R.R., Ashley D.C., Nicholson W.L. (2013). First Detection of Heartland Virus (Bunyaviridae: Phlebovirus) from Field Collected Arthropods. Am. J. Trop. Med. Hyg..

[42] Mayo Clinic Laboratories (2023). Test ID: HRTVS. Heartland Virus, RNA, Molecular Detection, PCR, Serum. https://www.mayocliniclabs.com/test-catalog/overview/620057#Performance.

[43] Basile A.J., Horiuchi K., Goodman C.H., Kosoy O., Panella A.J., Velez J.O., Pastula D.M., Brault A.C., Staples J.E., Calvert A.E. (2021). Development of diagnostic microsphere-based immunoassays for Heartland virus. J. Clin. Virol..

[44] Centers for Disease Control and Prevention (2023). For Healthcare Providers. Heartland Virus Disease (Heartland). https://www.cdc.gov/heartland-virus/healthcare-providers/index.html.

[45] Eichenauer D.A., La Rosée P. (2023). Treatment of hemophagocytic lymphohistiocytosis in patients in the intensive care unit. Inn. Med..

[46] Fill M.A., Compton M.L., McDonald E.C., Moncayo A.C., Dunn J.R., Schaffner W., Bhatnagar J., Zaki S.R., Jones T.F., Shieh W.J. (2017). Novel Clinical and Pathologic Findings in a Heartland Virus-Associated Death. Clin. Infect. Dis..

[47] Bratton R.L., Corey G.R. (2005). Tick-borne disease. Am. Fam. Physician.

[48] Monzón J.D., Atkinson E.G., Henn B.M., Benach J.L. (2016). Population and Evolutionary Genomics of *Amblyomma americanum*, an Expanding Arthropod Disease Vector. Genome Biol. Evol..

[49] Nielebeck C., Kim S.H., Pepe A., Himes L., Miller Z., Zummo S., Tang M., Monzón J.D. (2023). Climatic stress decreases tick survival but increases rate of host-seeking behavior. Ecosphere.

[50] Raney W.R., Perry J.B., Hermance M.E. (2022). Transovarial Transmission of Heartland Virus by Invasive Asian Longhorned Ticks under Laboratory Conditions. Emerg. Infect. Dis..

[51] Kwasnik M., Rola J., Rozek W. (2023). Tick-borne encephalitis—Review of the current status. J. Clin. Med..

[52] Crandall K.E., Millien V., Kerr J.T. (2023). Historical associations and spatiotemporal changes of pathogen presence in ticks in Canada: A systematic review. Zoonoses Public Health.

[53] Charles R.A., Bermudez S., Banovic P., Alvarez D.O., Diaz-Sanchez A.A., Corona-Gonzalez B., Etter E.M.C., Gonzalez I.R., Ghafar A., Jabbar A. (2021). Ticks and tick-borne diseases in Central America and the Caribbean: A One Health perspective. Pathogens.

[54] Cull B. (2022). Monitoring Trends in Distribution and Seasonality of Medically Important Ticks in North America Using Online Crowdsourced Records from iNaturalist. Insects.

[55] Jordan R.A., Egizi A. (2019). The growing importance of lone star ticks in a Lyme disease endemic county: Passive tick surveillance in Monmouth County, NJ, 2006–2016. PLoS ONE.

[56] Sagurova I., Ludwig A., Ogden N.H., Pelcat Y., Dueymes G., Gachon P. (2019). Predicted Northward Expansion of the Geographic Range of the Tick Vector *Amblyomma americanum* in North America under Future Climate Conditions. Environ. Health Perspect..

[57] Ma D., Lun X., Li C., Zhou R., Zhao Z., Wang J., Zhang Q., Liu Q. (2021). Predicting the Potential Global Distribution of *Amblyomma americanum* (Acari: Ixodidae) under Near Current and Future Climatic Conditions, Using the Maximum Entropy Model. Biology.

[58] Molaei G., Little E.A.H., Williams S.C., Stafford K.C. (2019). Bracing for the Worst—Range Expansion of the Lone Star Tick in the Northeastern United States. N. Engl. J. Med..

[59] Fujii H., Tani H., Egawa K., Taniguchi S., Yoshikawa T., Fukushi S., Yamada S., Harada S., Kurosu T., Shimojima M. (2022). Susceptibility of Type I interferon receptor knock-out mice to Heartland Bandavirus (HRTV) infection and efficacy of Favipiravir and Ribavirin in the treatment of mice infected with HRTV. Viruses.

[60] BioSpace MediVector Completes Patient Enrollment in Two Phase III Studies of Favipiravir for Influenza. 17 February 2015. https://www.biospace.com/article/releases/medivector-completes-patient-enrollment-in-two-phase-3-studies-of-favipiravir-for-influenza-/.

[61] Shah P.I., Orton C.M., Grinsztejn B., Donaldson G.C., Ramirez B.C., Tonkin J., Santos B.R., Cardoso S.W., Ritchie A.I., Conway F. (2023). Favipiravir in patients hospitalised with COVID-19 (PIONEER trial): A multicentre, open-label, phase 3, randomized controlled trial of early intervention versus standard care. Lancet Respir. Med..

[62] U.S. Food and Drug Administration (2022). Warning Letter. Extrapharmacy.Ru. Notice of Unapproved and Misbranded Drugs to United States Consumers over the Internet. MARCS-CMS 615132. https://www.fda.gov/inspections-compliance-enforcement-and-criminal-investigations/warning-letters/extrapharmacyru-615132-02152022.

[63] Toba S., Sato A., Kawai M., Taoda Y., Unoh Y., Kusakabe S., Nobori H., Uehara S., Uemura K., Taniguchi K. (2022). Identification of cap-dependent endonuclease inhibitors with broad-spectrum activity against bunyaviruses. Proc. Natl. Acad. Sci. USA.

[64] De Vlugt C., Sikora D., Pelchat M. (2018). Insight into influenza: A virus cap-snatching. Viruses.

[65] He X., Yang F., Wu Y., Lu J., Gao X., Zhu X., Yang J., Liu S., Xiao G., Pan X. (2023). Identification of tanshinone I as cap-dependent endonuclease inhibitor with broad-spectrum antiviral effect. J. Virol..

[66] Waters E.S., Kaiser E.E., Yang X., Fagan M.M., Scheulin K.M., Jeon J.H., Shin S.K., Kinder H.A., Kumar A., Platt S.R. (2021). Intracisternal administration of tanshinone IIA-loaded nanoparticles leads to reduced tissue injury and functional deficits in a porcine model of ischemic stroke. IBRO Neurosci. Rep..

[67] Davis S.L., Vazquez S. (2008). Anidulafungin: An evidence-based review of its use in invasive fungal infections. Core Evid..

[68] Szymański M., Chmielewska S., Czyżewska U., Malinowska M., Tylicki A. (2022). Echinocandins—Structure, mechanism of action and use in antifungal therapy. J. Enzym. Inhib. Med. Chem..

[69] Shen S., Zhang Y., Yin Z., Zhu Q., Zhang J., Wang T., Fang Y., Wu X., Bai W., Dai S. (2022). Antiviral activity and mechanism of the antifungal drug, anidulafungin, suggesting its potential to promote treatment of viral diseases. BMC Med..

[70] Mendoza C.A., Yamaoka S., Tsuda Y., Matsuno K., Weisend C.M., Ebihara H. (2021). The NF-κB inhibitor, SC75741, is a novel antiviral against emerging tick-borne bandaviruses. Antivir. Res..

[71] Ogawa M., Shimojima M., Saijo M., Fukasawa M. (2021). Several catechins and flavonols from green tea inhibit severe fever with thrombocytopenia syndrome virus infection in vitro. J. Infect. Chemother..

[72] U.S. Food and Drug Administration (2018). Understanding Unapproved Use of Approved Drugs “Off Label”. https://www.fda.gov/patients/learn-about-expanded-access-and-other-treatment-options/understanding-unapproved-use-approved-drugs-label.

[73] Yoshikawa T. (2021). Vaccine Development for Severe Fever with Thrombocytopenia Syndrome. Viruses.

[74] Matsuno K., Orba Y., Maede-White K., Scott D., Feldmann F., Liang M., Ebihara H. (2017). Animal Models of Emerging Tick-Borne Phleboviruses: Determining Target Cells in a Lethal Model of SFTSV Infection. Front. Microbiol..

[75] Suleman M., Balouch A.R., Randhawa A.Y.W., Khan T., Muddhassir M., Ullah A., Jan A.U., Zia M.A., Ali S.S., Khan A. (2022). Characterization of proteome wide antigenic epitopes to design proteins specific and proteome-wide ensemble vaccines against heartland virus using structural vaccinology and immune simulation approaches. Microb. Pathog..

[76] Wang Y., Griffiths A., Brackney D.E., Verardi P.H. (2022). Generation of Multiple Arbovirus-like Particles Using a Rapid Recombinant Vaccinia Virus Expression Platform. Pathogens.

